# Purpura as a late complication of covid-19 infection that should not be ignored: Case report and brief review

**DOI:** 10.1016/j.amsu.2021.103216

**Published:** 2022-01-01

**Authors:** Meryem Jabri, Nour El Houda Lamaasab, Chaimae Daoudi, Fadoua Jabrouni, Hajar Benzekri, Amine Bouchlarhem, Nourdinne Oulali

**Affiliations:** a Faculty of Medicine and Pharmacy, Mohammed I^st^ University, Oujda, Morocco; b Department of Emergency, Mohammed VI University Hospital Mohammed I University Oujda, Morocco

**Keywords:** Vascular purpura, COVID-19, Late complication, Cutaneous manifestations

## Abstract

**Introduction and importance:**

The SARS COV2 infection is a challenging pandemic that has affected millions of people with a very high mortality rate. In addition to the typical respiratory symptoms, it can also cause variable skin lesions, such as vascular purpura in some exceptional cases.

**Case presentation:**

We report the case of a 60-year-old woman who was admitted for a SARS COV2 infection, the evolution was marked by the appearance of a vascular purpura at D20 after the beginning of the symptoms.

**Discussion:**

The cutaneous manifestations associated with the SARS COV2 infection are polymorphic. Vascular purpura is one of them. Its diagnosis is retained in the light of a combination of arguments, which makes it a real challenge for the physician to diagnose it. The management of the disease is based on a symptomatic treatment. The clinical evolution is, in general, favorable.

**Conclusion:**

Although rare and still not fully explained, skin involvement during SARS COV2 infection has been described. It should not be neglected and it should be diagnosed early and treated appropriately, especially in asymptomatic patients.

## Highlights

• Infection with the COVID-19 virus can be manifested by clinical signs of varying severity, sometimes fatal.
• Being a systemic disease, SARS COV2 infection may be revealed, in addition to respiratory symptoms, by skin lesions including vascular purpura
• We report a case of vascular purpura occurring post-infection

## 1 Introduction

The covid-19 pandemic made a huge impact on world health and was one of the most important challenges faced by the World Health Organization. This virus is responsible for several symptoms with varying degrees of severity, ranging from acute respiratory distress syndrome (ARDS), which can be life-threatening, to skin disorders, including vascular purpura. We report the case of a 60-year-old woman admitted suffering from vascular purpura 20 days after having been infected by SARS-COV2.

## 2 Case presentation

We report the case of a 60-year-old female patient, with a history of hypertension under a well-controlled calcium channel blocker, admitted to the emergency room with exertional dyspnea associated with an influenza-like illness that had been evolving for a week. The general examination, on admission, found a conscious patient, dyspneic with a SpO2 = 92% at AA, polypneic with a respiratory rate at 22cpm, without cyanosis or signs of respiratory distress, hemodynamically stable with a PAS at 140mmhg and a PAD at 79mmhg and a heart rate at 88 bpm, afebrile with a temperature at 38.2°, with a weight of 85 kg and a height of 168 cm as well as a BMI = 35 kg/m2.

The pleuropulmonary examination found asymmetric basithoracic crackling rales. The rest of the examination was normal.

- Considering the pandemic specific circumstances, a PCR was performed and came back positive. The CT scan without contrast injection showed peripheral ground glass lesions typical of SARS VOC 19 infection with an estimated 20% involvement. Because of her clinical stability, the patient was treated as an outpatient with the following national anti-COVID protocol: Azithromycin 500 mg at D1, then 250mg from D2 to D7, Vitamin C 1000 mg/12H for 15 days, Vitamin D 100000 IU/week, Zinc 45mg/day for 15 days, with regular medical follow-up. The initial evolution was favorable with a clear clinical improvement.

**Fig. 1:**
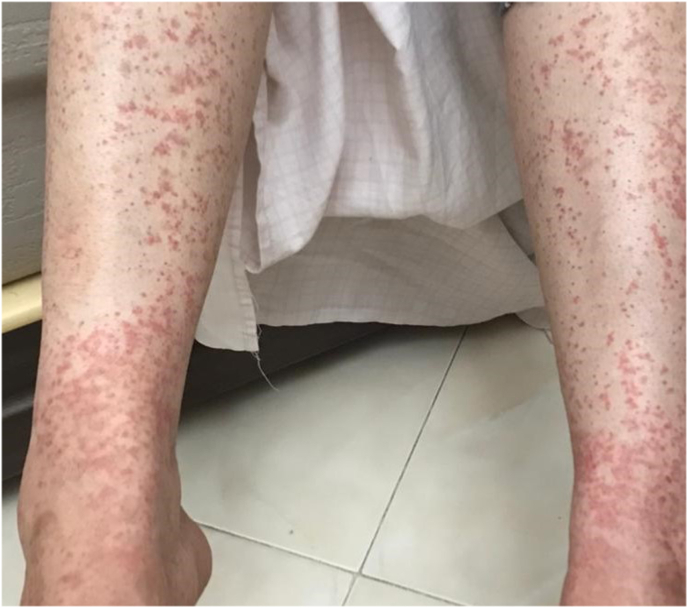
Skin lesions made of punctiform erythematous macules.

**Fig. 2:**
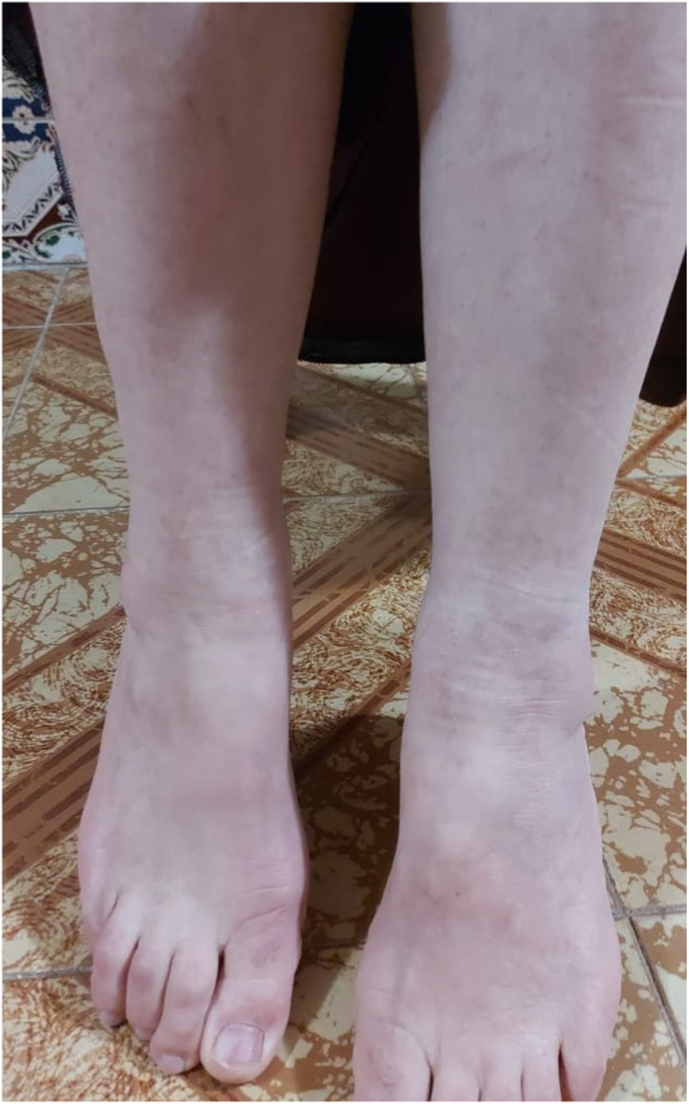
Disappearance of skin lesions.

After 20 days since the beginning of her symptoms, the patient presented skin lesions made of punctiform erythematous macules that did not fade with in vitro pressure on the lower limbs (Fig. 1), with progressive extension without signs of infection or other associated signs. A biological assessment was requested. The blood count showed a correct platelet count of 440,000/mm3 with the rest of the blood lines normal. Bleeding time, partial thromboplastin time and prothrombone levels were normal. The rest of the hemosatosis workup was without abnormalities. During the etiological investigation, the patient had no signs of infection other than COVID-19, no signs of infective endocarditis, no signs of drug use, and neither clinical sign pointing to systemic vasculitis such as arthralgia, ocular or renal involvement. There was no evidence of rheumatoid purpura, AL amyloidosis or diabetes. Therefore, the diagnosis of post-covid vascular purpura was retained. The patient was put on antihistamines and topical emollients. The evolution was marked by the progressive disappearance of the purpuric lesions after one week (Fig. 2).

## 3 Discussion

Since the emergence of covid 19 virus, the state of the global health has been deeply affected due to the severity, rapidity of spread, and high risk of morbidity and mortality of this infection [1], which makes it the greatest challenge that the world health organization has faced. SARS-COV2 belongs to the β-CoVs family, it mainly uses the angiotensin-converting enzyme receptor (ACE 2) to enter cells. This receptor is mainly expressed in the lungs, which explains the pulmonary tropism of the virus [2], but also in the skin, which explains the diversity of the clinical picture. In a very simple way, the pathophysiology of organ damage in COVID19 infection is explained by the abnormal response of the host against this infection by the overexpression of inflammatory cytokines of free radicals and also chemokines, it is indeed the cytokine release syndrome (CRS) [3]. This CRS will itself explain the acute respiratory distress syndrome which is the first cause of mortality.

It is important to highlight that COVID19 is a general disease that can be complicated by several events: thromboembolic complications that represent a real challenge for the clinician to manage and which are explained by the state of hypercoagulability secondary to the inflammatory storm, cardiovascular complications are also a real problem to manage [4,5], and given the overexpression of the ACE2 receptor in several organs, the attacks can be multiple and represented in some cases by the skin attack.

Skin involvement remains rare and is described in different countries, ranging from 20.4% of patients (18 of 88) in an Italian cohort to 1.8% (2 of 1099 patients) in a Chinese cohort [6]. The rash is dominated by erythematopapular lesions, exceptionally purpura. According to a study by Magro [7] only 2 cases of vascular purpura have been reported in the literature. Several mechanisms are involved depending on the type of skin involvement; the role of interleukin 6 in the stimulation of inflammatory cells, direct activation of complement by the virus causing purpura by a thrombotic mechanism through their deposits, increased production of interferon alpha secondary to a type I interferenopathy induced by the virus [8]. Post-covid vascular purpura is clinically manifested by erythematous lesions that do not erase under in vitro pressure, infiltrated with a predominantly sloping appearance that spares the mucous membranes. These lesions are often diffuse and asymptomatic [8] and often occur several weeks after infection with SARS-CoV-2 or concomitantly.

The positive diagnosis of post-covid vascular purpura is based on the clinical presentation, normal blood count and bleeding time, and skin biopsy with an often-positive covid PCR. Regarding the prognosis of these patients, there is no correlation between the severity of SARS-COV 2 infection and skin involvement [9]. However, the discovery of vascular purpura in a context of covid should lead to the search for systemic signs that could be life-threatening [10]. Management remains mainly symptomatic, and relies on antihistamines in case of severe pruritus or even dermocorticoids with disappearance of cutaneous signs in the majority of cases without complication.

The SCARE guildlines were used in the writing of this paper [11].

## 4 Conclusion

Although rare, skin involvement is one of the manifestations of Covid-19 that must be known for early detection of the infection, especially in asymptomatic patients, in order to limit the spread of this global pandemic. However, the causal link between SARS-COV2 and skin involvement remains poorly elucidated.

## Ethical approval

The ethical committee approval was not required give the article type (case report). However, the written consent to publish the clinical data of the patients was given and is available to check by the handling editor if needed.

## Sources of funding

None.

## Author contribution

Meryem Jabri: study concept or design, data collection, data analysis or interpretation, writing the paper. Nour El houda Lamassab: Data collection, data analysis, writing the paper. Chaimae Daoudi: Data collection, data analysis. Fadoua Jabrouni: Data collection, data analysis. Hajar Benzekri: Data collection, data analysis. Amine Bouchlarhem: Data collection, Data analysis. Nouredine Oulali: supervision and data validation.

## Consent

Written informed Consent was obtained from the patients for publication of this case report and accompanying images. A copy of the written consent is available for review by the Editor-in-Chief of this journal on request.

## Registration of research studies

This is not an original research project involving human participants in an interventional or an observational study but a case report. This registration is was not required.

## Guarantor

Meryem Jabri.

Nour El Houda Lamassab.

## Declaration of competing interest

None.
